# BerkSEL: A scale-invariant laser beyond the Schawlow-Townes two-mirror strategy

**DOI:** 10.1038/s41467-024-46338-0

**Published:** 2024-03-06

**Authors:** Boubacar Kanté

**Affiliations:** 1 Department of Electrical Engineering and Computer Sciences, University of California Berkeley, Berkeley, CA 94720 USA; 2 Materials Sciences Division, Lawrence Berkeley National Laboratory, Berkeley, CA USA

**Keywords:** Semiconductor lasers, Nonlinear optics

## Abstract

I argue that a surface emitting laser that remains single mode irrespective of its size, a scale-invariant laser, should of necessity also waste light at the edge. This is a fundamental departure from the Schawlow-Townes two-mirror strategy that keeps light away from mirrors and edges to preserve gain and minimize loss. The strategy was implemented in the recent discovery of the Berkeley Surface Emitting Laser (BerkSEL).

## All existing lasers use the Schawlow-Townes two-mirror strategy

The feedback in laser systems has been provided by a two-mirror strategy (that can take various forms), envisioned by Charles Townes more than six decades ago^1^. Light oscillates back and forth between the two mirrors to be amplified with as little loss as possible, owing to the very small optical gain available in early lasers. The two-mirror strategy has been used to construct all existing lasers. Unfortunately, this strategy amplifies multiple modes when the size of the cavity increases. The two-mirror strategy leads to high order gaussian modes. I argue that conventional mirrors (including photonic bandgap reflectors, be they trivial or topological) make it impossible for lasers to remain single mode as the mode size grows. I introduce a new strategy enabled by the counter-intuitive, but intentional, replacement of mirrors by lossy boundaries. Loss is permissible, since we are in a new era in which optical gain has become abundant and ubiquitous, particularly with semiconductor gain media. Single mode behavior requires bringing light all the way to a cavity boundary in a strategy that wastes photons at the edge as the price for large single mode area. Additionally, there is a counter-intuitive necessity for Dirac-cone, zero-gap band dispersion to achieve single mode performance.

**Fig. 1:**
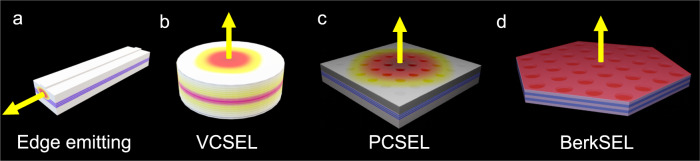
Beyond Schawlow-Townes two-mirror cavities to scale single-mode volume. Semiconductor lasers, including an edge emitting laser (**a**), a vertical cavity surface emitting laser (VCSEL) (**b**), and a photonic crystal surface emitting laser (**c**), all operating on gaussian-like modes. These lasers use the original Schawlow-Townes two-mirror strategy that allows higher order modes to set in. **d** The scale-invariant Berkeley Surface Emitting Laser (BerkSEL) brings the lasing mode to lossy cavity edges, at variance with the Schawlow-Townes two-mirror strategy. Spatial hole burning is eliminated in BerkSELs, and higher order mode lasing is forbidden to allow single-mode lasing.

Figure 1a–c illustrate common semiconductor lasers including edge emitting lasers, vertical cavity surface emitting lasers (VCSELs), and photonic crystal surface emitting lasers (PCSELs)^2–6^. Fascinating progress has been made with these platforms that are now used in many applications including consumer electronics, virtual reality, interconnects in data centers, manufacturing, defense, imaging, or medicine to cite a few. Nonetheless, they all use the original Schawlow-Townes two-mirror strategy which stingily maximizes gain and minimizes losses of the lasing modes in the cavities. For all existing semiconductor lasers (Fig. 1a–c), the field in the aperture decays exponentially in directions perpendicular to the emission direction (yellow arrow). Edge emitting lasers of Fig. 1a operate on very high order longitudinal modes and are limited by catastrophic optical damage. Further increasing their power would require increasing the transverse dimensions of the waveguide, thus unifying the strategy with surface emission (Fig. 1b, c). The Schawlow-Townes two-mirror strategy allows higher order modes to take advantage of the residual gain near the mirrors and edges of the cavity. In the Schawlow-Townes two-mirror strategy, the fundamental optical mode has high intensity at the center of the cavities and has low intensity at the boundaries near the mirrors. In this strategy, optical gain is thus depleted at the center of the cavity but not at the edges. Higher order modes take the opportunity to sneak in around the boundaries and mirrors benefiting from the undepleted gain. A fundamental mode that oscillates in the laser cavity (aperture) cannot entirely deplete the gain available in the cavity aperture because the field must decay around the mirrors and cavity edges, allowing higher order parasitic modes to build up.

## Discovery of scale-invariant semiconductor lasers

The promise of lasers has always been coherence, i.e., to concentrate all the laser power into a single spatial mode. This is in addition to temporal coherence that refers to sharp spectral linewidth. Since Schawlow-Townes’ invention of lasers, a single spatial mode has been achieved by limiting the effective size of the optical gain region. A larger optical gain region, i.e., a larger distance between the mirrors, leads to multiple spatial modes. The undesired additional higher order modes can be suppressed by limiting the size of the laser gain region, but this comes at the expense of laser power. Sixty-five years after the Schawlow-Townes’ idea, optical cavity strategy has not changed. The cavity modes have been optimized and engineered to minimize losses but at the risk of allowing higher order modes.

Owing to their simplicity, semiconductor lasers have become ubiquitous, and each one is smaller than a grain of sand. Correspondingly, their laser power is limited to a few Watts at most. A fundamental question is whether we can construct larger, higher-power single mode lasers by abandoning the Schawlow-Townes two-mirror strategy.

The optical gain in the original realization of lasers in the 1960’s was very small. Indeed, scientists in the early 1960’s doubted the laser diode was possible due to the tiny fraction of electrical energy released as light when current carriers recombined in a semiconductor. As a result, the first diode lasers cavities had linear dimensions at least two orders of magnitude longer than current single mode VCSELs (Vertical Cavity Semiconductor Lasers). The volume was thus about six orders of magnitude larger than current VCSELs^2^. Optical gain was new in the 1960s, precious, and very weak. The laser mode had to be well-confined, kept away from edges where light could escape. As a result, there was little lasing power to saturate that gain around the edges. These peripheral gain regions create an opening for higher order modes to set in, defeating the desired single-mode performance. The situation has significantly changed since the 1960s. Optical gain is no longer weak and precious thanks to a better understanding of direct-gap semiconductors. The solution is to allow a desired single mode to escape all the way, into all edges, sweeping up all residual gain, preventing the emergence of higher order modes as shown in Fig. 1d^7^. Indeed, an oscillatory envelope signifies that the wavevector is finite in the cavity. To bring the field into all edges and enable scale-invariance, oscillatory modes must be prevented. This requires a fundamentally different quantization in the cavity to enable a mode with zero wavevector. The Dirac-cone together with appropriate boundaries, enables such quantization. The open-Dirac cavity, the cavity at the heart of BerkSELs, enables the required unconventional quantization^7^. Its fundamental mode, with zero momentum, does not oscillate and naturally brings the full optical field to the edges of the cavity, by uniformly occupying the entire aperture. This works, but then the single-mode light would experience escape losses, possibly overwhelming the precious gain. Today, we can indeed afford a single optical mode which has losses at exterior edges. Since the goal is large area, or large volume single-mode lasing, we shall note the larger the mode, the less significant are the edge losses. The proposed new mechanism successfully depletes the entire gain region, right up to the edges, preventing a second optical mode from emerging^7^. The open-Dirac geometry is needed to make the constant-intensity mode (designed to suffer from some losses at the edges) to win the mode competition against finite wave-vector modes (that experience a depleted gain medium).

This new insight and approach solve a more than six-decade challenge by going beyond the standard Schawlow-Townes style optical cavity and recognize that modern semiconductor gain materials have an excess of gain that can be employed for other purposes, such as assuring single-mode operation^7^. The new solution produces a new type of scale-invariant lasing mode that was surprisingly never predicted nor observed in any wave physics system. The insight will lead to powerful and efficient lasers for industrial materials processing, for communication networks, for military applications, which could also provide a laser propulsion for small, unmanned spacecraft, such as the Starshot program^8^ or semiconductor lasers for fusion promising bountiful and carbon-free energy^9^. The new strategy also provides a unique insight into dynamical systems in the longstanding search for robust physics and architectures that can synchronize a very large number of oscillators beyond models such as the Kuramoto model^10^.
